# Tobacco-Users

**Published:** 1868-08

**Authors:** 


### TOBACCO-USERS.

It has become very common to invest chewing-tobacco and snuff in lead-foil. Herr Hockel examined some snuff from a quantity, part of which had been used by a patient who was laboring under a severe attack of lead poisoning, and found that it contained two and a half per cent of metallic lead. The tobacco near the corners of the package, being more perfectly inclosed by the foil, contained the most lead, which is decomposed by dampness and remains in the tobacco or snuff in the form of carbonate of lead, which is the white lead paint of commerce, which inflicts such horrible sufferings on many of those whose business compels them to work in it. The slaves of the disgusting "weed" would do well to make a note of this, and either abandon the inexcusable filthiness or avoid using any that is enveloped with lead-foil.

